# Retrospective Cohort Study of Practical Applications of Paramagnetic Seed Localisation in Breast Carcinoma and Other Malignancies

**DOI:** 10.3390/cancers14246215

**Published:** 2022-12-16

**Authors:** Céline Clement, Lieve Schops, Ines Nevelsteen, Soetkin Thijssen, Chantal Van Ongeval, Machteld Keupers, Renate Prevos, Valerie Celis, Patrick Neven, Sileny Han, Annouschka Laenen, Ann Smeets

**Affiliations:** 1 Multidisciplinary Breast Centre Department Surgical Oncology, University Hospitals Leuven, KU Leuven, 3000 Leuven, Belgium; 2 Department Gynaecology, Noorderhart Ziekenhuis Pelt, 3900 Pelt, Belgium; 3 Multidisciplinary Breast Centre Department Radiology, University Hospitals Leuven, KU Leuven, 3000 Leuven, Belgium; 4 Multidisciplinary Breast Centre Department of Gynaecology and Obstetrics, University Hospitals Leuven, KU Leuven, 3000 Leuven, Belgium; 5 Leuven Biostatistics and Statistical Bioinformatics Centre (L-BioStat), 3000 Leuven, Belgium

**Keywords:** paramagnetic seed localisation, breast conserving surgery

## Abstract

**Simple Summary:**

Paramagnetic seeds are a safe alternative for the wire-guided localisation of non-palpable breast lesions. This retrospective, multicentre review confirms the feasibility of magnetic seed localisation, as well as the higher risk for positive margins in cases of breast carcinoma with associated DCIS. Soft tissue lesions and lymph nodes associated with other malignancies, e.g., melanoma, can also be localised with paramagnetic seeds. This offers perspectives for future applications, such as for the de-escalation of axillary treatment in breast cancer.

**Abstract:**

(1) Background: Paramagnetic seeds are a safe alternative for the wire-guided localisation of non-palpable breast lesions, but can also be applied for non-breast lesions. This study presents the experience with a paramagnetic seed, MagSeed^®^ (Endomagnetics Ltd., Cambridge, UK, CE-registered and FDA-cleared), in an academic and non-academic breast centre. (2) Methods: Multicentre, retrospective analysis of 374 consecutive patients who underwent surgery after paramagnetic seed localisation (MSL) between 2018 and 2020. Indications for localisation included non-palpable breast lesions (*n* = 356), lymph nodes (*n* = 15) or soft tissue lesions (*n* = 3). The primary outcome was feasibility and the rate of positive section margins. The secondary outcome was predictive factors for positive section margins. (3) Results: The accurate excision of high-risk breast lesions, lymph nodes and soft tissue lesions was seen in 91.07% (*n* = 56). Positive section margins were observed in 7.86% (*n* = 25) after breast conserving surgery for invasive or ductal carcinoma in situ (DCIS) (*n* = 318). Invasive breast cancer associated with DCIS (*p* = 0.043) and the size of DCIS (*p* < 0.001) were significantly correlated with the positive section margins. (4) Conclusion: This study confirms the feasibility of MSL, as well as the higher risk for positive margins in cases of breast carcinoma with associated DCIS. Soft tissue lesions and lymph nodes associated with other malignancies, e.g., melanoma, can also be localised with paramagnetic seeds. This offers perspectives for future applications, such as the de-escalation of axillary treatment in breast cancer.

## 1. Introduction

Screening mammography has increased the diagnosis of non-palpable invasive breast cancer lesions, ductal carcinoma in situ (DCIS) and BIRADS-3 breast lesions [1,2,3,4]. Therefore, a reliable method to localise these lesions peri-operatively is necessary [5]. To date, wire-guided localisation has been the golden standard; however, this method has several disadvantages, such as technical difficulties and patient and surgeon comfort. In addition, it requires a same day procedure on the day of operation at the radiology department to avoid the dislocation of the wire [6]. Paramagnetic Seed Localisation (MSL) has been proven to be a safe and effective method to localise non-palpable breast lesions, while addressing several limitations of wire-guided localisation (WGL) such as the decoupling of radiology and surgery planning [7,8,9,10,11,12]. There are limitations to MSL, such as a limited ability to use MRI when a paramagnetic seed is implanted due to scattering on MRI, up to four centimetres [6].

In addition, the inability to reposition the seed after deployment and the need for adjusted (nonferromagnetic) surgical instruments are the main limitations of these non-radioactive seeds, such as Magseed ^®^.(Endomagnetics Ltd., Cambridge, UK, CE- and FDA-registered).

Beyond its use in breast tissue, paramagnetic seeds can be employed to localise axillary lymph nodes, for instance in targeted axillary dissection (TAD) after neo-adjuvant chemotherapy, or soft tissue lesions [13,14]. Besides breast carcinoma, paramagnetic seeds have been used to localise metastases of melanoma [15].

Paramagnetic seed localisation was the standard of care for all breast conserving surgery in our tertiary referral hospital, since 2017, and since 2018 in our secondary care hospital, after previously using wire-guided localisation [16].

In this article, we present the results of a retrospective multi-centre cohort study regarding the oncological safety of MSL in non-palpable breast lesions, the feasibility of localising non-palpable locoregional breast cancer recurrences and lymph node metastasis for breast cancer and other types of malignancies.

## 2. Materials and Methods

The data were retrospectively collected between 1 September 2018 and 30 April 2020 at a tertiary care hospital (UZ Leuven) and a secondary care hospital (Noorderhart Ziekenhuis Pelt). All patients who received a Magseed^®^ for non-palpable breast lesions, lymph nodes and soft tissue lesions were included. A total of 374 patients (respectively, 324 and 50 patients per institute) underwent a localisation procedure with a total of 379 seeds. The data were separately analysed for soft tissue and breast tissue applications, and separately for the two hospitals.

Patient characteristics, details from the seed placement procedure and surgical procedure, possible complications and the histopathological results were determined from the electronic medical records. MRI was performed preoperatively at the discretion of the clinician and systematically in cases of lobular breast cancer. All seeds were placed under ultrasound guidance at the radiology department with, if applicable, verification of the correct positioning with mammography. Lesions of the breast that were not visible on ultrasound, e.g., microcalcifications, were localised with the aid of a temporary wire during placement. If the Magseed^®^ was placed >5 mm from the lesion, the placement was defined as not accurate. If a patient underwent neo-adjuvant systemic therapy, an MRI-compatible titanium marker clip was placed prior to the start of the systemic therapy. A marker was also placed if the patient underwent a stereotactic biopsy or vacuum assisted biopsy. In these cases, a new mammography was performed to check for clip migration. When there was no clip migration, a seed was placed in proximity to the marker prior to surgery. When there was migration of the marker, a seed was placed in the correct position with the aid of a temporary wire. The seeds were subsequently retrieved in the operating room by using the Sentimag^®^ (Endomag) magnetometer and the retrieval was verified by detecting the seed in the specimen. A specimen radiograph was obtained to ensure the visualization of the seed in the resection specimen and to assess the section margins for the microcalcifications in both centres. Postoperative hematoma, surgical site infection or complication during the placement of the seed were recorded if stated in the clinical notes. Following breast conserving surgery, all patients with invasive breast cancer or ductal carcinoma in situ received radiotherapy and, if applicable, adjuvant systemic therapy.

The outcomes were: (1) oncological safety defined as free margins (‘no ink on tumour’) after lumpectomy for invasive and non-invasive breast carcinoma (R0-resection) and (2) need for reoperation or (3) rate of intraoperative selective shaving of a specific margin within the remaining cavity. In addition, the safety and feasibility of localising the lymph node metastasis for breast cancer and other types of malignancies, as well as soft tissue lesions, was assessed.

Statistical analysis was performed with SAS (version 9.4 of the SAS System for Windows). Descriptive statistics were used to generate means, medians and percentages. A Student’s *t*-test and a Chi-square test were applied for the assessment of the associations. Bland-Altman analysis was carried out to compare the discrepancies between the size on preoperative radiological examinations and postoperative measurements on the pathology specimen of the tumour. *p* values that were less than 0.05 were considered significant.

Ethical approval was granted by the Institutional Review Board.

## 3. Results

A total of 374 patients were included, with 379 seeds placed. In five patients with a non-palpable breast lesion, a seed was placed bilaterally. Overall, 318 (85.02%) patients underwent MSL for an invasive breast carcinoma or ductal carcinoma in situ, 38 patients (10.13%) for a high-risk or benign breast lesion and 18 patients (4.81%) for a soft tissue lesion or lymph node.

Accurate placement occurred in 95.99% (359 patients) of the cases. No migration of the seeds was detected. All of the Magseeds^®^ were placed under ultrasound guidance. In 12 patients (3.20%) with microcalcifications of the breast, a temporary wire was placed to aid placement by ultrasound. Complications during the placement of the seed included hematoma in six patients (1.60%) and three cases of unintentionally placing multiple seeds due to a malfunction of the cartridge (0.80%).

All of the seeds were retrieved, except in one case, with a soft-tissue tumour in the lower extremity. The mean interval between placement of the seed and operative retrieval was nine days (range 0–312 days). Intraoperative complications included anaphylactic shock after patent blue injection in one patient and the dislocation of the Magseed^®^ from the specimen in one patient. Postoperative complications were observed in 5.09% of the patients (16 patients), consisting of hematoma (9 patients) and surgical site infection or delayed wound healing (7 patients). Postoperative hematoma was more frequently seen after Vacuum Assisted Biopsy (VAB) was performed preoperatively (*p* = 0.027, 0.77% vs. 6.38%).

### 3.1. Invasive Breast Cancer and Ductal Carcinoma In Situ

**Figure 1:**
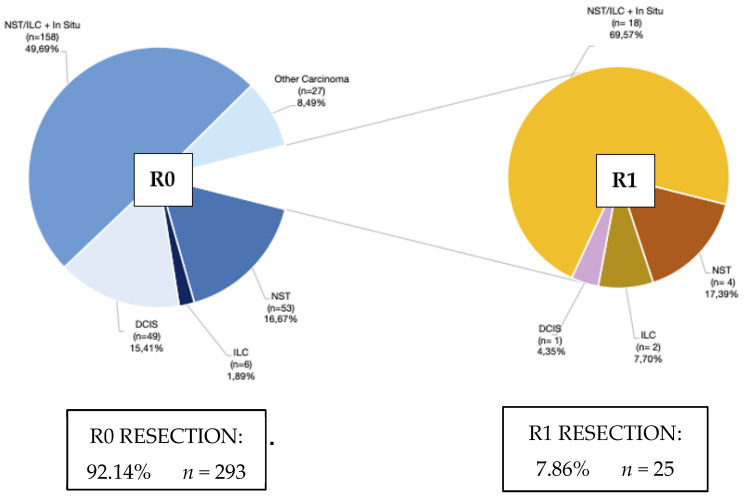
Pathological characteristics of patients with invasive breast cancer and ductal carcinoma in situ: R0 versus R1 resection. Abbreviations: NST = Invasive Carcinoma of No specific Type, ILC = Invasive Lobular Carcinoma, Other Invasive Carcinoma: Mucinous, Apocrine, Tubular, Papillary, Metaplastic, Neuro-endocrine subtypes.

In 318 patients, a paramagnetic marker was used to localise a non-palpable invasive breast carcinoma or DCIS for breast conserving surgery. The patient demographics are listed in Table 1. The majority of these lesions were screen-detected (67.29%) and preoperative MRI was performed in 38.99%. Nine patients (3.36%) were initially diagnosed with a BIRADS-3 lesion on core biopsy; however, this was proven to be an invasive breast cancer of DCIS upon postoperative final pathology. Positive section margins were observed in 25 patients (7.86%, centre 1; 7.09% and centre 2 12.00%). The pathological results are listed in Figure 1. Upon the final pathology of the specimen, no residual malignant tissue was found in the specimen in 5.34% of cases, due to complete pathological response after neo-adjuvant therapy or due to the complete resection that occurred with core needle biopsy, preoperatively.

Specimen radiography confirmed the retrieval of the seed in all cases, with the exception of one case in which the seed was dislocated from the specimen, intraoperatively. In 98.42% of cases, the seeds were present in the first specimen after lumpectomy. In five patients (1.58%), an intraoperative selective shaving of a specific margin within the remaining cavity was necessary during primary surgery to obtain the seed. Shaving intraoperatively was performed in 27.04% (86 patients) of the cases, following the radiological or macroscopic pathological evaluation of the specimen. There was no significant difference in the shaving rates between centre 1 (25.37%) and centre 2 (36.00%). In 82.55% of cases, the shaving specimen contained no further malignant tissue. In 13 cases (15.11%), there was a positive margin after shaving. The presence of DCIS did not influence the shaving rates significantly (*p* > 0.05).

The association of invasive with in situ malignancy significantly increased the incidence of positive section margins (*p* = 0.043). In patients with positive section margins, this was due to the transection of the invasive component in 60.00% of cases.

Furthermore, the size of the DCIS is associated with a higher risk for transection: the mean size of the DCIS on final pathology was 17.80 mm if the margins were negative and 30.13 mm if the margins were positive (*p* < 0.001).

Of the six patients with positive section margins in centre 2, five (83.33%) underwent intraoperative shaving. Therefore, in centre 2, as opposed to centre 1, a significant association was seen between the need for intraoperative shaving and positive section margins on definitive postoperative pathology (*p* = 0.018). No correlation was observed between the affected margins sides and positive margins (*p* = 0.097) or affected breast quadrant (*p* = 0.631). No association was observed between positive section margins and neo-adjuvant therapy, Body Mass Index, subtype of breast cancer (ductal vs. lobular) or the type of preoperative radiological investigation. Bland-Altman analysis showed that the discrepancy between the size in preoperative radiological examinations and the postoperative measurement on the pathology specimen of the tumour is low (*p* = 0.854).

After positive section margins, either additional surgery (10 patients) or adjuvant radiotherapy including a higher boost dose (15 patients) was performed. Ten patients (3.14%, centre 1 2.23% centre 2 8.00%) underwent additional surgery after initial breast conserving surgery, which consisted of mastectomy in eight patients and re-lumpectomy in two patients.

### 3.2. Benign and BIRADS-3 Breast Lesions

In 38 cases, a paramagnetic marker was used to localise a benign (26.32%) or high-risk lesion BIRADS-3 (73.68%) of the breast. The patients’ characteristics can be found in Table 2. In total, 94.74% of these lesions were non-palpable. Nine patients out of the 47 patients initially diagnosed with a benign or high-risk lesion in the core biopsy proved to be an invasive breast cancer or DCIS in the final postoperative pathology.

### 3.3. Soft Tissue Lesions and Lymph Nodes

In 18 cases, a paramagnetic marker was used to localise a soft tissue lesion (33.33%, six patients) or a lymph node (66.67%, 12 patients) for breast cancer (14 patients) and other malignancies. The patients’ characteristics can be found in Table 3. Seven cases concerned a recurrence of breast carcinoma during follow-up, in either a regional lymph node (71.43%, five patients) or in the pectoralis muscle (28.57%, two patients). All of the seeds could be retrieved, with the exception of one case with a soft-tissue tumour in the lower extremity, due to the absence of a percutaneous signal intraoperatively to localise the Magseed^®^. All four of the invasive lesions concerning the pectoralis muscle could be retrieved with negative section margins. The lymph nodes were located in the axilla, infraclavicular or intramammary.

## 4. Discussion

In this cohort, 96.86% of patients with invasive and non-invasive breast carcinoma were adequately treated with breast conserving surgery with MSL and only ten patients (3.14%) underwent reoperation after the initial procedure with positive resection margins. Therefore, our data support the previous findings, although with lower reoperation rates [8].

We observed several factors that are significantly associated with positive resection margins. Whilst, in the analysis conducted by Lamb et al., age and the mode of biopsy (ultrasound vs. mammography vs. MRI) were associated with a higher risk of positive section margins in cases of DCIS and breast conserving therapy, this study could not confirm these findings [18]. However, the presence of DCIS in association with invasive malignancy significantly correlates with positive resection margins. (*p* = 0.043) Similar to this finding, the size of DCIS in the postoperative specimen was associated with positive section margins (*p* < 0.001). This is comparable to the findings for WGL or radioactive seed localisation [19,20,21,22]. The determining component for section margins is not solely DCIS, as in 60% of the cases with positive section margins this was due to the invasive component. There was no association between neo-adjuvant systemic therapy and positive margin rates, in contrast to the previous literature [23].

In this cohort, the preoperative size estimation in the radiological investigations matched with the pathological findings. Lam et al. recently published that a preoperative MRI increases negative resection margins in patients with DCIS [24].

However, a preoperative MRI did not influence the presence of positive section margins in this cohort, and may, therefore, not be sufficient to assess the presence of DCIS, as previously reported in the literature [25,26]. This could be due selection bias, as only 38.99% of our patients with breast cancer or DCIS underwent MRI. If extensive DCIS is suspected during the preoperative radiological investigations or in the core needle biopsy, patients should be counselled for a higher risk of a positive section margin.

Several studies have confirmed that paramagnetic seed localisation is easy to implement [11,27,28,29,30]. When comparing the results of a tertiary referral centre with a high volume of cases to the results in the first 50 patients in a secondary care hospital, we observed no significant differences, with the exception of one. The need for intraoperative shaving was associated with positive section margins in centre 2, but not in centre 1. A possible explanation for this difference might be the diversity of radiological and/or pathological assessments between the two centres due to interobserver variability. The rate of intraoperative shaving was comparable to the literature [31]. In the majority of cases, this additional tissue did not contain any remaining invasive or in situ carcinoma. In 13 cases out of 86 patients with invasive breast carcinoma or DCIS who underwent intraoperative shaving (15.12%), there was a positive margin after shaving. Intraoperative shaving and the immediate radiological assessment of the surgical margins have been proven to be useful for reducing the positive margin rates and are therefore recommended in the updated American Society of Breast Surgeons Toolbox [31,32,33,34].

In addition to breast tissue, this study confirms the feasibility of MSL for the resection of axillary and intramammary lymph nodes, even after previous surgery of the breast or axilla, given that half of these cases concerned a breast cancer recurrence [14]. This offers perspectives for the de-escalation of axillary management e.g., TAD after neo-adjuvant therapy in breast carcinoma, currently investigated in several ongoing trails [35]. Lymph node metastases of other malignancies, such as melanoma and ovarian cancer, can also be localised.

In cases of solitary breast cancer metastasis in the pectoralis muscle, paramagnetic markers can be useful when performing a modified mastectomy with a targeted partial resection of the pectoralis muscle.

In this cohort, only one soft tissue lesion of the lower extremity could not be excised. Intraoperatively, the percutaneous signal of the paramagnetic marker could not be detected due to interference of the metal components of the operation table and the location of the lesion in the upper part of the thigh. This case serves as an example that paramagnetic seed localisation has its limitations.

We report few complications, comparable to previous studies. Although observed in a small number, a significantly higher incidence of postoperative hematoma after vacuum-assisted biopsy (*p* = 0.027) was observed [7,8,9,10,11,29,30]. For current practice, the placement of the paramagnetic marker is advised to take place after the partial resorption of the VAB-associated hematoma.

Limitations of this study include the retrospective method and multi-centre set-up, potentially creating heterogeneity in surgical, pathological and radiological techniques. In addition, every patient was included following the implementation of MSL in both centres. Therefore, we may observe a learning curve; however, this is not significant in Magseed^®^ procedures, as reported in previous studies [11].

## 5. Conclusions

MSL has been proven to be a safe and effective localisation method for non-palpable breast lesions. The retrospective results of a high-volume tertiary referral centre, compared to the first 50 results of a secondary care hospital, were comparable. Therefore, this retrospective cohort study confirms previous findings. However, this article warrants attention for the increased risk of positive margins in cases of DCIS, consistent with wire-guided wide local excision.

Paramagnetic seeds can be reliably used to localise breast lesions, metastatic soft tissue lesions and lymph nodes, tp also for nodal metastatis from other malignancies than breast carcinoma. This offers perspectives for future applications, such as in targeted axillary dissection for the de-escalation of axillary treatment in breast cancer.

## Figures and Tables

**Table 1:** Baseline characteristics of patients with invasive breast cancer and ductal carcinoma in situ.

Characteristic	UZ LEUVEN		PELT		TOTAL	
	*n* = 268	84.28%	*n* = 50	15.72%	*n* = 318	100%
Age (years) (mean, range)	60.79	(33–84)	58.94	(30–83)	60.50	(30–84)
Menopausal Status						
Premenopausal	43	16.04%	14	28.00%	57	17.92%
Perimenopausal	17	6.34%	0	0.00%	17	5.34%
Postmenopausal	208	77.61%	36	72.00%	244	76.72%
Screen detected lesion	177	66.04%	37	74.00%	214	67.30%
Preoperative MRI	92	34.33%	32	64.00%	124	38.99%
Neo-adjuvant therapy	28	10.45%	17	44.00%	45	14.15%
Chemotherapy (+/− targeted therapy)	26	9.70%	17	44.00%	43	13.52%
Endocrine therapy	2	0.75%	0	0.00%	2	0.63%
Estrogen Receptor Positive	242	90.30%	39	58.00%	281	88.36%
HER 2 Neu Receptor Positive	25	9.32%	12	24.00%	37	11.64%
Pathology Subtype						
NST	36	13.43%	21	42.00%	57	17.92%
ILC	4	1.49%	4	8.00%	8	2.52%
DCIS	46	17.16%	4	8.00%	50	15.72%
NST/ILC + in situ	156	58.20%	20	40.00%	176	55.35%
Other	26	9.70%	1	2.00%	27	8.49%
Tumor Grade						
Grade 1	73	27.24%	15	30.00%	88	26.67%
Grade 2	140	52.24%	17	34.00%	157	49.37%
Grade 3	55	20.52%	18	36.00%	73	22.96%
Clinal Nodal Stage						
cN+	14	5.22%	4	8.00%	18	5.66%
pT (TNM- classification [17])						
pTis	45	16.79%	5	10.00%	50	15.72%
pT1	158	58.96%	41	82.00%	199	62.58%
pT2	37	13.80%	4	8.00%	41	12.89%
pT3	1	0.37%	0	0.00%	1	0.31%
ypT (TNM-classification [17])						
ypT0	8	2.99%	7	14.00%	15	4.72%
ypTis	5	1.87%	2	4.00%	7	2.20%
ypT1	12	4.48%	2	4.00%	14	4.40%
ypT2	3	1.12%	1	2.00%	4	1.26%

Abbreviations: NST = Invasive Carcinoma of No specific Type, ILC = Invasive Lobular Carcinoma, Other Invasive Carcinoma: Mucinous, Apocrine, Tubular, Papillary, Metaplastic, Neuro-endocrine subtypes.

**Table 2:** Baseline characteristics of patients with benign or B3 breast lesions on core biopsy.

Characteristic	*n* = 47	
Age (years) (mean, range)	54.83	(36–82)
Screen detected lesion (*n*, %)	33	70.21%
Size of lesion on pathology specimen (mm) (mean, range)	10.98	(1–30)
Weight pathology specimen (gram) (mean, range)	12.26	(5–65)
Pathology on resection specimen (*n*, %)		
Atypical Ductal Hyperplasia	6	12.77%
Isolated small cell LCIS	6	12.77%
Intraductal papilloma	18	38.30%
Fibroadenoma	3	6.38%
PASH	3	6.38%
Other (radial scar, hemangioma, sclerosing adenosis)	3	6.38%
Invasive breast cancer or DCIS	9	19.15%

**Table 3:** Baseline characteristics of patients with resected soft tissue lesions and lymph nodes.

Characteristic	*n* = 17	
Age (years) (mean, range)	56.78	(31–88)
Lymph Nodes (*n*, %)		
Breast Cancer	10	58.85%
Ovarian Cancer	1	5.88%
Melanoma	1	5.88%
Soft Tissue Lesion (*n*, %)		
Nodular fasciitis Pectoralis Muscle	1	5.88%
Breast Cancer Pectoralis Muscle	4	23.53%
Recurrence Breast Cancer (*n*, %)	7	41.18%
Neo-adjuvant therapy (*n*, %)	7	41.18%

## Data Availability

The data presented in this study are available on request from the corresponding author.
